# Respiratory and Immune Response to Maximal Physical Exertion following Exposure to Secondhand Smoke in Healthy Adults

**DOI:** 10.1371/journal.pone.0031880

**Published:** 2012-02-15

**Authors:** Andreas D. Flouris, Giorgos S. Metsios, Andres E. Carrill, Athanasios Z. Jamurtas, Polychronis D. Stivaktakis, Manolis N. Tzatzarakis, Aristidis M. Tsatsakis, Yiannis Koutedakis

**Affiliations:** 1 FAME Laboratory, Institute of Human Performance and Rehabilitation, Centre for Research and Technology, Thessaly, Greece; 2 Department of Research and Technology Development, Biomnic Ltd., Trikala, Greece; 3 School of Sports, Performing Arts and Leisure, University of Wolverhampton, Wolverhampton, United Kingdom; 4 Department of Exercise Sciences, University of Thessaly, Trikala, Greece; 5 Centre of Toxicology Science and Research, School of Medicine, University of Crete, Crete, Greece; University of Texas at Tyler, United States of America

## Abstract

We assessed the cardiorespiratory and immune response to physical exertion following secondhand smoke (SHS) exposure through a randomized crossover experiment. Data were obtained from 16 (8 women) non-smoking adults during and following a maximal oxygen uptake cycling protocol administered at baseline and at 0-, 1-, and 3- hours following 1-hour of SHS set at bar/restaurant carbon monoxide levels. We found that SHS was associated with a 12% decrease in maximum power output, an 8.2% reduction in maximal oxygen consumption, a 6% increase in perceived exertion, and a 6.7% decrease in time to exhaustion (*P*<0.05). Moreover, at 0-hours almost all respiratory and immune variables measured were adversely affected (*P*<0.05). For instance, FEV_1_ values at 0-hours dropped by 17.4%, while TNF-α increased by 90.1% (*P*<0.05). At 3-hours mean values of cotinine, perceived exertion and recovery systolic blood pressure in both sexes, IL4, TNF-α and IFN-γ in men, as well as FEV_1_/FVC, percent predicted FEV_1_, respiratory rate, and tidal volume in women remained different compared to baseline (*P*<0.05). It is concluded that a 1-hour of SHS at bar/restaurant levels adversely affects the cardiorespiratory and immune response to maximal physical exertion in healthy nonsmokers for at least three hours following SHS.

## Introduction

Thirty years after the publication of the first evidence regarding the unfavorable health effects of secondhand smoke (SHS) exposure [1] more than half of American, European and Chinese adult nonsmokers suffer daily SHS as the prevalence rates of smoking continue to rise across the globe [2]. Moreover, current estimates suggest that 80% of American and 76% of European teenagers are regularly exposed to SHS [3], [4]. Major chronic diseases are generally the result of long-term processes, yet recent data by our group [5], [6], [7], [8] and others [9], [10], [11] show that even brief SHS exposures appear to initiate mechanisms that contribute to disease pathogenesis. For instance, shortly after being exposed to SHS there is a marked increase in interleukin (IL) 1 beta [6], [12], IL4, tumor necrosis factor alpha (TNF-α) [12], white blood cell count, C-reactive protein, homocysteine, fibrinogen [11] as well as leukocyte counts accompanied by an immune cell activation [13]. Interestingly, the observed inflammatory reactions persist for at least 3 hours following SHS exposure [12] and appears to be more potent in men compared to women [6], [12].

The SHS-induced immune response raises concerns for potentially intensified system disruption when additional strains are added such as physical exertion, especially in individuals with (or at risk for) cardiovascular disease, chronic lung disease or allergies. Indeed, it is not uncommon for individuals to suffer SHS at home or at the workplace and subsequently to exert significant physical effort, such as walk fast for several minutes or climb a few sets of stairs while carrying heavy objects. For instance, blue-collar workers (e.g., construction labors, factory workers, freight handlers, assemblers, mechanics) that are constantly active, reaching high energy expenditures (e.g., an 86-kg individual tending a furnace in a steel mill utilizes on average 648 kcal·h^−1^) [14], are more likely to be exposed to workplace SHS compared to white-collar workers [15], [16], [17].

Understanding the acute and short-term effects of SHS on the cardiorespiratory and immune response to physical exertion is essential because the physical and metabolic adaptations involved can increase the risk of acute coronary complications and life-threatening myocardial ischemia even in apparently healthy individuals [18], [19]. Therefore, we conducted a randomized single-blind crossover experiment to assess the cardiorespiratory and immune response to maximal physical exertion prior to as well as 0, 1, and 3 hours following 1-hour of moderate SHS.

## Materials and Methods

### Ethics Statement

The study was conducted according to the principles expressed in the Declaration of Helsinki and was approved by the University of Thessaly Ethics Review Board.

### Experimental Design

Recruitment was conducted through newspaper and radio announcements, wall posters, as well as word of mouth. Exclusion criteria (assessed via medical history) included: smoking, pregnancy, evidence of cardiac or pulmonary disease, and previous disease or medications known to affect lung function. All women participants were premenopausal with regular menstruation and were tested during the late luteal phase of their menstrual cycle.

**Figure 1:**
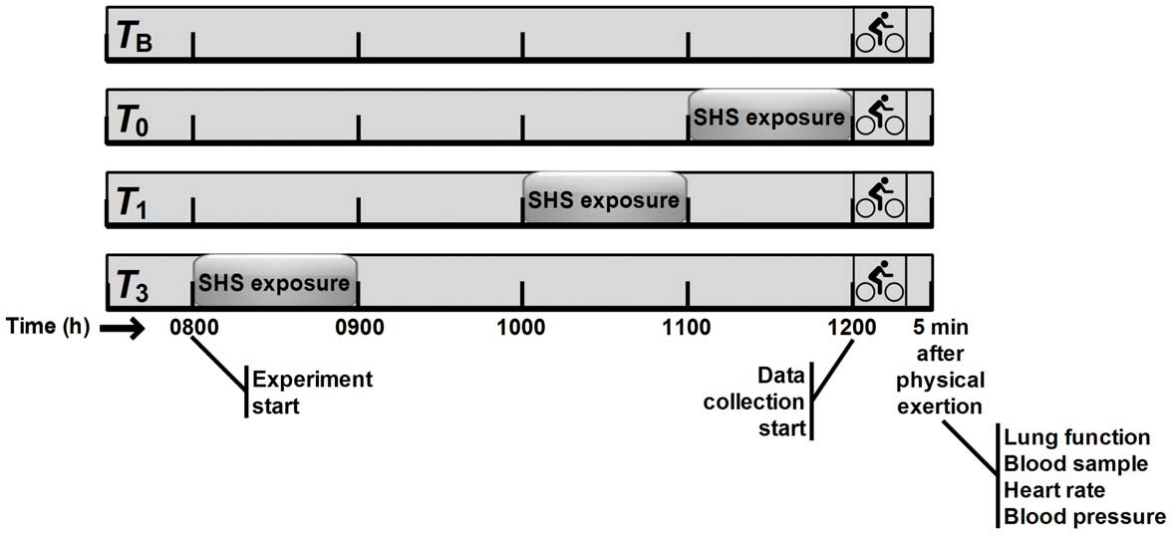
Overview of the experimental protocol. In the baseline trial (*T*_B_) participants underwent a physical exertion bout that started at 1200 h without any SHS. In the remaining trials, one hour of SHS was administered at either 0800 h (*T*_3_), 1000 h (*T*_1_) or 1100 h (*T*_0_), while the same physical exertion protocol initiated at 1200 h. SHS = secondhand smoke.

All participants were given a detailed verbal description of the protocol, followed by extensive familiarization with all data collection procedures and instruments during a familiarization visit performed >5 days prior to testing. The experimental protocol is illustrated in Figure 1. To eliminate the effect of cotinine's comparatively long half-life (i.e., approx. 20 hours [20]), the study adopted a randomized crossover design with participants visiting the laboratory on four different occasions, separated by ≥7 days, where they underwent four different trials in a random order through a random allocation algorithm (PASW Statistics 18, SPSS Inc., Chicago, USA). Furthermore, in order to eliminate the effect of diurnal variation, all data were collected at the same time of the day. Participants arrived in the laboratory at 0730 h for every trial. During the baseline trial participants underwent a physical exertion bout that started at 1200 h without any SHS. In the remaining trials, one hour of SHS was administered either at 0800 h, or at 1000 h or at 1100 h, while the same physical exertion protocol initiated always at 1200 h. This design enabled data collection in response to maximal physical exertion without SHS (*T*_B_) as well as immediately following (*T*_0_), at one hour after (*T*_1_) and at three hours after SHS (*T*_3_), with the data being collected at the same time of the day and without the effect of previous or subsequent physical exertion since only one assessment was conducted per trial. Five minutes following the end of physical exertion (passive recovery with participants lying in semi-supine position), heart rate, arterial blood pressure, cotinine, lung function and cytokine levels were evaluated as described below.

Measurements of the targeted variables were conducted using identical pre-calibrated equipment in a quiet room maintained at 22–25°C air temperature. The same room was used for the participants to remain at rest before or after SHS and until the initiation of physical exertion. For all trials, caloric intake was restricted while *ad libitum* water consumption was permitted. All participants arrived at the laboratory following a 10-hour fast, and were instructed to refrain from strenuous physical exertion and other excessive stressors for 72 h prior to each trial. All testing was conducted by the same trained investigators, who were unaware of the specific trial that each participant was undergoing. To ensure that the investigators would not be able to differentiate between trials, all participants were given a shirt and athletic pants to wear upon exiting the chamber following all exposure trials and prior to the physical exertion bout in the baseline trial. These clothes had been previously exposed to tobacco smoke and emitted strong tobacco scent. Moreover, since the physical exertion protocol was conducted always at 1200 h (with SHS at 0800 h, or at 1000 h or at 1100 h), it was not possible for the investigators collecting the data to know which trial a subject was undergoing.

#### Secondhand Smoke (SHS)

During *T*_0_, *T*_1_ and *T*_3_, participants remained seated at rest for 1 hour inside a 6×5×4 m environmentally controlled chamber (air temperature: 24°C; air velocity: 0.05 m•s^−1^; humidity: 45%). The SHS was adjusted at a carbon monoxide (CO) concentration of 23±1 ppm to meet levels previously reported for bar/restaurant environments [21] checked via continuous measurement by a Horiba (MEXA-311GE) CO-CO_2_ analyzer. The desired CO concentration was achieved by combustion of cigarettes from various popular brands (i.e., equal number of Camel, Davidoff Classic, Gauloises Filter, Original Red Lucky Strike, Marlboro Reds, Prince Classic and Silk Cut Purple King Size cigarettes). The cigarettes were lit and placed on ashtrays to burn until reaching the filter at different places in the chamber, while fans were used to ensure circulation throughout the chamber. When the desired CO concentration was attained, the participant entered the chamber.

#### Serum and Urine Cotinine

Serum and urine cotinine were measured. Both serum and urine samples were obtained to ensure validity since, although urine is the specimen of choice for SHS (urine cotinine concentration is ∼10× higher than in circulation), such samples may be subject to contamination. Whole blood (15 ml) was collected by a certified phlebotomist from an antecubital vein into plain evacuated test tubes and a portion of it (5 ml) was used for serum cotinine analysis. Immediate sample processing included allowing the blood to clot at room temperature (i.e., ∼23°C) for 30 min and centrifugation at 1000× *g* for 10 min. The serum layer was then removed and frozen in multiple aliquots at −20°C until analyzed. For urine cotinine analyses, 80 mL urine void was collected in polyethylene specimen jars (Fisher Scientific, Pittsburgh) and was immediately frozen at −20°C, until analyzed.

The serum sample preparation of cotinine biochemical analysis included mixture of an aliquot (1 ml) of each sample with 1 ml of buffer solution (pH = 6.88). The urine sample preparation included mixture of an aliquot (5 ml) of each sample with 2.5 ml of buffer solution (pH = 6.88). All analyses were conducted via electron ionization mass spectrometric confirmatory analysis using a Finnigan Mat GCQ_TM_ system equipped with an HP-5MSI (30 m×0.25 mm ID×0.25 µm) capillary column (J&W Scientific). Two microliters of each sample were injected into the system in the splitless mode. Analysis conditions were as follows: The column temperature program started from 90°C for 1 min and was raised to 280°C at the rate of 20°C·min^−1^. The injector temperature was 280°C. The transfer line temperature was set at 300°C. The mass spectrometer acquisition parameters were: Ion source 200°C, electron impact ionization 70eV and electron multiplier voltage of 1200V. The mass spectrometer was operated at the selected ion-monitoring mode and was programmed for the detection of m/z = 84 for nicotine (cotinine) and m/z = 180, 209 for ketamine. Under these conditions nicotine (cotinine) eluted at 6.16 min and ketanine at 10.15 min.

#### Lung Function

Forced vital capacity (FVC), forced expiratory volume in 1 second (FEV_1_), FEV_1_/FVC ratio, peak expiratory flow (PEF) and maximum expiratory flow when 75%, 50% and 25% of FVC remains in the lungs (MEF_75%_, MEF_50%_, MEF_25%_, respectively) were measured in a single-expiration maneuver via spirometry. In addition, maximum voluntary ventilation, respiratory rate, and tidal volume were measured over a 15-second time period and data were extrapolated to 1-minute values. The lung function assessment measurements were conducted using a hand held spirometer (Spiromed 180, Fukuda Sangyo, Tokyo) calibrated before each use and conformed to the American Thoracic Society recommendations [22]. The percent of predicted FEV_1_ (_%_FEV_1_) was also calculated based on sex- and age-specific reference values [23].

#### Cytokine Assessment

Levels of specific pro-inflammatory [IL-6, TNF-α and interferon gamma (IFN-γ)] and allergic-related [IL-4 and IL-5] cytokines were assessed using 10 ml of whole blood following blood collection and immediate sample processing identical to that previously described for serum cotinine. Cytokine levels were measured using commercially available enzyme-immunosorbent assay kits (Biosource Europe S.A.) according to the manufacturer's protocol. The lower limits of detection were: IL-4 and IL-5: 5 pg·mL^−1^, IL-6: 2 pg·mL^−1^, TNF-α: 3 pg·mL^−1^, and IFN-γ: 5 pg·mL^−1^. The reproducibility was assessed by two consecutive measurements on the same day in samples from a subgroup of 15 individuals. The coefficient of variation for the assessed cytokines were: IL-4: 6.7%, IL-5: 3.8%, IL-6: 3.7%, TNF-α: 3.6%, and IFN-γ: 6.6%.

#### Physical Exertion Protocol and Recorded Variables

Participants underwent a maximal oxygen uptake protocol consisting of a 3-minute warm-up period of steady-state cycling (Monark Ergomedic 839E, Vansbro, Sweden) at 60 W, followed by increments of 30W·min^−1^ until exhaustion. Pedaling rate was maintained at 60 revolutions·min^−1^ throughout. An automated gas analyzer (Vmax 29, Sensormedics, USA) was used to record respiratory variables every 20 sec while individuals inspired room air. The highest oxygen uptake (ml·kg^−1^·min^−1^) for any 20-sec interval was recorded as the individual's maximal oxygen uptake. The percent predicted maximal oxygen uptake [24] and percent predicted power output [25] were calculated based on published norms for average cardiorespiratory fitness. During the physical exertion protocol, respiratory exchange ratio and mean power output (in kJ) were continuously recorded, while subjective ratings of perceived exertion (RPE) [ranging from 0 (not hard at all) to 20 (very very hard) with increments of 1] [26] were measured every five minutes.

Five minutes following the end of physical exertion (passive recovery with participants lying in semi-supine position), heart rate (Polar Electro, Kempele, Finland), arterial blood pressure, cotinine, lung function and cytokine levels were evaluated. Blood pressure measurements were conducted by the same trained investigator using the same mercury sphygmomanometer (AS007, UK). The arterial blood pressure readings were used to calculate mean arterial pressure (i.e., diastolic+[0.333·(systolic-diastolic)]) [27].

### Statistical Analysis

The data were divided by sex into groups given the known sexual dimorphism in the acute SHS effects [6]. Sample size calculations were conducted based on values before and after a similar 1-hour SHS exposure for serum cotinine (61.9±18.8 vs. 187.0±76.3 in men and 67.5±23.6 vs. 185.3±66.1 in women) and mean work done [242.4±61.1 vs. 135.6±41.2 in men and 159.0±38.3 vs. 84.8±26.6 in women) from a previous SHS experiment in our laboratory [28] (see also correction [29]). The resulting minimum required total sample size (two-tailed hypothesis) for a statistical power level of 0.8 and a probability level of 0.05 was 10.

Given that several variables did not fulfill the assumptions of multivariate analysis of variance, non-parametric statistical techniques were employed. Two Kruskal-Wallis ANOVA were computed separately for men and women to assess the effect of time (i.e., *T*_B_, *T*_0_, *T*_1_ and *T*_3_) after SHS on all the examined variables. These analyses were followed by sex-specific post-hoc Mann–Whitney U tests assessing differences between time points (i.e., *T*_B_ vs. *T*_0_; *T*_0_ vs. *T*_1_; *T*_1_ vs. *T*_3_; *T*_B_ vs. *T*_1_; and *T*_B_ vs. *T*_3_). Comparisons between sexes for the same time point were conducted using Mann–Whitney U tests. The sample size calculations were conducted with PASS 2000 (Hintze J. Number Cruncher Statistical Systems, Kaysville, UT, USA) software, while all other statistical analyses were carried out with PASW Statistics. The level of significance was set at *P*<0.05.

## Results

Sixteen healthy adults [8 men (28.3±4.4 years; BMI: 22.7±2.2 kg/m^2^), 8 women (26.2±3.9 years; BMI: 21.3±1.1 kg/m^2^)] from the general population volunteered and provided informed consent prior to participation. The subjects were generally active individuals, but were not participating in organized sport activities, while some of them had participated in a previous secondhand smoke experiment by our group [28]. Results showed that, compared to *T*_B_, there were marked differences in the majority of the examined variables at *T*_0_ while values at *T*_1_ and – mainly – at *T*_3_ had returned to *T*_B_ levels. In men, Kruskal-Wallis ANOVA revealed that IL-4 (*P* = 0.002), TNF-α (*P* = 0.002), perceived exertion (*P* = 0.017), recovery systolic blood pressure (*P* = 0.012), as well as serum (*P* = 0.021) and urine cotinine (*P*<0.001) increased whereas the FEV_1_/FVC (*P* = 0.019) decreased across time. The same analysis in women demonstrated that TNF-α (*P* = 0.001), perceived exertion (*P* = 0.003), recovery systolic blood pressure (*P* = 0.025), recovery mean arterial pressure (*P* = 0.039), respiratory rate (*P*<0.001), as well as serum (*P* = 0.006) and urine (*P*<0.001) cotinine increased across time. In contrast, maximal oxygen consumption (*P* = 0.023), percent predicted power output (*P* = 0.047), FEV_1_ (*P* = 0.048), _%_FEV_1_ (*P* = 0.001), FEV_1_/FVC (*P*<0.001), MEF_50%_ (*P*<0.001), MEF_25%_ (*P*<0.001), maximum voluntary ventilation (*P* = 0.046), and tidal volume (*P* = 0.004) decreased across time.

Results from sex-specific post-hoc t-tests are presented in Tables 1, 2, 3. A general trend was observed whereby marked differences were detected between *T*_B_ and *T*_0_ while values in *T*_1_ and – mainly – *T*_3_ showed a tendency towards returning to *T*_B_ levels. The majority of variables demonstrated statistically significant results, yet some did not mainly due to increased dispersion. Regarding performance, perceived exertion was increased and percent predicted maximal oxygen uptake was decreased in both sexes following SHS. In men, absolute and relative maximum power output was decreased, and in women maximal oxygen uptake was reduced while maximal respiratory exchange ratio was elevated (*P*<0.05). The cardiovascular indices assessed revealed increased recovery systolic and mean arterial blood pressure following SHS in both sexes (*P*<0.05). Moreover, maximum systolic and diastolic blood pressure during physical exertion were also elevated only in women (*P*<0.05). The lung function parameters measured showed that FEV_1_/FVC was decreased following SHS in both sexes (*P*<0.05). Interestingly, women demonstrated significant reductions (*P*<0.05) in all other lung function parameters measures, with the exception of PEF. With respect to immune function, TNF-α and IFN-γ were increased following SHS in both sexes, as well as IL-4 only in men (*P*<0.05). At *T*_3_, most of the assessed variables had returned to *T*_B_ levels (*P*>0.05). However, mean values of perceived exertion, recovery systolic blood pressure, as well as serum and urine cotinine at *T*_3_ remained different than *T*_B_ levels (*P*<0.05) in both sexes. Moreover, significant differences between *T*_3_ and *T*_B_ (*P*<0.05) were observed for IL4 and TNF-α in men, as well as for exercise systolic blood pressure, _%_FEV_1_, FEV_1_/FVC, respiratory rate and tidal volume in women.

**Table 1:** Mean ± SD of cardiorespiratory variables for men and women for the statistically significant post-hoc comparisons.

		*T*_B_	*T*_0_	*T*_1_	*T*_3_
_Max O2 uptake_	M	42.2±5.5	38.8±5.2	39.3±5.5	39.4±4.5
(ml·kg^−1^·min^−1^)	W	39.6±2.3	36.3±1.7^a^	36.5±1.7^b^	36.8±3.4
_% Max O2 uptake_	M	102.4±8.5	94.1±8.1^a^^, ^ c	95.4±12.6	95.8±10.9
(%)	W	111.9±8.0	102.7±7.5^a^^, ^ c	103.3±7.1	104.2±12.2
Max respiratory	M	1.31±0.06^c^	1.35±0.08	1.34±0.07	1.34±0.10^c^
exchange ratio	W	1.22±0.06^c^	1.28±0.08^a^	1.27±0.06	1.22±0.08^c^
Max Power	M	300.0±45.4^c^	251.3±27.5^a^^, ^ c	270.0±27.8^c^	285.0±35.9^c^
(kJ)	W	224.5±48.2^c^	207.7±36.2^c^	217.0±44.9^c^	217.5±51.0^c^
_%_Max Power	M	123.6±14.2	104.2±14.9^a^^, ^ c	111.9±14.3^c^	117.5±10.9
(%)	W	146.1±38.2	135.1±30.5^c^	141.6±38.8^c^	141.8±40.9
Perceived Exertion	M	17.9±1.1	19.1±1.0^a^	18.9±0.9	19.0±0.9^b^
	W	18.2±0.4	19.2±0.3^a^	19.0±0.9^b^	19.3±0.3^b^
Time to exhaustion	M	10∶36±1∶40	9∶23±0∶57	9∶38±0∶52	9∶52±0∶47
(min:sec)	W	9∶59±0∶22	9∶47±0∶24	9∶54±0∶27	9∶44±0∶25
Max HR	M	185.0±7.7	185.4±7.2	185.4±7.0	184.9±4.5
(beats·min^−1^)	W	183.4±6.1	184.4±6.4	184.5±5.7	184.3±4.4
Recovery HR	M	112.4±13.9	115.4±4.1^c^	112.9±9.8	112.3±6.4^c^
(beats·min^−1^)	W	102.9±15.1	108.4±2.7^c^	106.9±8.3	102.9±6.9^c^
Max SBP	M	173.8±10.8	184.1±17.7	186.0±13.7	183.4±12.1
(mmHg)	W	173.0±5.0	180.4±16.4^a^	182.2±10.5^b^	181.7±7.4^b^
Recovery SBP	M	119.3±6.5	131.0±4.8^a^	132.0±6.7^b^	129.4±8.4^b^
(mmHg)	W	126.8±14.7	136.1±11.9^a^	137.6±10.2^b^	136.3±11.5^b^
Max DBP	M	81.8±6.3	87.8±9.8	87.9±7.6	83.8±10.1
(mmHg)	W	81.2±5.8	90.1±9.0^a^	89.8±5.3^b^	86.2±7.9
Recovery DBP	M	71.3±6.9	75.0±7.6	73.3±8.1	72.8±9.7
(mmHg)	W	74.6±5.0	78.5±4.1	76.9±3.6	75.8±5.5
Max MAP	M	87.2±4.9	93.7±5.0	92.8±6.2	91.6±7.1
(mmHg)	W	92.0±6.8	97.7±4.9	97.1±5.3	95.9±5.6
Recovery MAP	M	87.2±4.9	93.6±5.0^a^	92.8±6.2^b^	91.6±7.1
(mmHg)	W	92.0±6.8	97.7±4.8^a^	97.1±5.3^b^	95.9±5.6

Note:

a = statistically significant (P<0.05) difference from previous measurement.

b = statistically significant (P<0.05) difference of *T*_1_ or *T*_3_ from *T*_B_.

c = statistically significant (P<0.05) difference between sexes for the same measurement.

Key: _%_Max O_2_ uptake: percent predicted maximal oxygen uptake; HR: heart rate; SBP, DBP, and MAP: systolic, diastolic and mean arterial blood pressure, respectively; M: men; W: women.

**Table 2:** Mean ± SD of cotinine and lung function for men and women for the statistically significant post-hoc comparisons.

		*T*_B_	*T*_0_	*T*_1_	*T*_3_
Serum Cotinine	M	9.4±4.3	22.5±9.9^a^	34.1±18.3^b^	28.7±20.1^b^
(ng·mL^−1^)	W	8.1±2.8	27.0±8.8^a^	33.6±19.3^b^	28.8±13.6^b^
Urine Cotinine	M	82.3±12.6	169.2±51.2^a^	273.7±102.7^a^	262.1±93.6^b^
(ng·mL^−1^)	W	74.5±14.3	165.1±56.6^a^	249.4±93.2^a^^, ^ b	250.6±97.8^b^
FVC	M	5.4±1.1^c^	5.5±1.1^c^	5.4±1.1^c^	5.4±1.1^c^
(L)	W	3.7±0.5^c^	3.7±0.6^c^	3.8±0.6^c^	3.6±0.6^c^
FEV_1_	M	5.1±0.7^c^	4.2±0.8^c^	4.5±0.8^c^	4.6±0.7^c^
(L)	W	3.5±0.3^c^	2.9±0.4^a^^, ^ c	3.0±0.4^b^^, ^ c	3.2±0.4^c^
_%_FEV_1_	M	114.8±12.6	98.3±15.0	103.3±15.5	105.3±13.4
(%)	W	110.1±4.5	93.0±6.8^a^	95.4±5.8^b^	102.1±8.4^b^
FEV_1_/FVC	M	0.94±0.12	0.79±0.06^a^	0.83±0.08^b^	0.86±0.09
	W	0.94±0.05	0.79±0.03^a^	0.80±0.04^b^	0.89±0.04^a^^, ^ b
PEF	M	10.3±1.4^c^	10.2±1.3^c^	9.7±1.3^c^	10.4±1.4^c^
(L·sec^−1^)	W	8.0±1.7^c^	8.0±1.7^c^	7.5±1.5^c^	8.2±1.8^c^
MEF_75%_	M	8.4±1.6	7.1±1.1^c^	7.6±1.2^c^	8.4±1.4
(L·sec^−1^)	W	7.1±1.1	5.4±1.1^a^^, ^ c	6.2±1.2^c^	6.7±1.4
MEF_50%_	M	5.6±1.4	4.6±1.1	4.9±1.0^c^	5.5±1.1
(L·sec^−1^)	W	5.0±0.4	3.6±0.5^a^	4.0±0.5^b^^, ^ c	4.6±0.6^a^
MEF_25%_	M	3.1±1.4	2.0±1.3	2.5±1.2	3.0±1.4
(L·sec^−1^)	W	3.5±0.3	2.7±0.5^a^	2.6±0.3^b^	3.2±0.3^a^
MVV	M	139.0±22.7^c^	113.6±38.2	120.3±33.1	129.7±25.8
(L)	W	116.6±18.3^c^	88.0±20.7^a^	97.2±16.0	109.4±17.5
RR	M	133.8±41.4^c^	162.3±59.2	154.2±59.8	154.3±52.8
(#)	W	101.9±10.2^c^	158.5±11.9^a^	146.2±14.9^a^^, ^ b	139.3±19.1^b^
TV	M	1.17±0.48	0.98±0.66	0.95±0.61	1.08±0.56
(L)	W	1.17±0.22	0.81±0.25^a^	0.70±0.14^b^	0.87±0.20^a^^, ^ b

Note:

a = statistically significant (P<0.05) difference from previous measurement.

b = statistically significant (P<0.05) difference of *T*_1_ or *T*_3_ from *T*_B_.

c = statistically significant (P<0.05) difference between sexes for the same measurement.

Key: FVC: forced vital capacity; FEV_1_: forced expiratory volume in 1 second; _%_FEV_1_: percent predicted FEV_1_; PEF: peak expiratory flow; MEF_75%_, MEF_50%_, MEF_25%_: maximum expiratory flow when 75%, 50% and 25% of FVC remains in the lungs, respectively; MVV: maximum voluntary ventilation; RR: respiratory rate; TV: tidal volume; M: men; W: women.

**Table 3:** Mean ± SD of cytokine production for men and women for the statistically significant post-hoc comparisons.

		*T*_B_	*T*_0_	*T*_1_	*T*_3_
IL4	M	29.0±9.4	47.4±7.1^a^^, ^ c	47.6±11.4^b^^, ^ c	43.8±6.7^b^^, ^ c
(pg·mL^−1^)	W	30.6±8.7	32.3±8.0^c^	30.9±8.4^c^	31.1±11.9^c^
IL5	M	102.5±91.5	167.7±180.7	152.8±197.1	143.6±157.6
(pg·mL^−1^)	W	112.5±99.4	163.2±93.4	157.1±72.8	147.2±76.6
IL6	M	3.6±2.4	5.1±3.9	6.0±3.9	6.6±3.9
(pg·mL^−1^)	W	11.7±17.6	15.8±21.4	18.7±23.1	20.3±24.2
TNF-α	M	7.8±2.6	14.5±2.0^a^	15.1±3.7^b^^, ^ c	14.8±1.9^b^^, ^ c
(pg·mL^−1^)	W	6.7±1.7	13.1±2.4^a^	10.2±2.3^b^^, ^ c	8.5±2.0^c^
IFN-γ	M	0.45±0.22	0.88±0.25^a^	0.82±0.27^b^	0.80±0.22^b^
(pg·mL^−1^)	W	0.47±0.29	0.92±0.32^a^	0.78±0.42	0.89±0.50

Note:

a = statistically significant (P<0.05) difference from previous measurement.

b = statistically significant (P<0.05) difference of *T*_1_ or *T*_3_ from *T*_B_.

c = statistically significant (P<0.05) difference between sexes for the same measurement.

Key: IL4, 5 and 6: interleukins 4, 5 and 6, respectively; TNF-α: tumor necrosis factor alpha; IFN-γ: interferon gamma; M: men; W: women.

## Discussion

Previous results by our group [5], [6], [7], [8] in resting nonsmokers showed that brief SHS at bar/restaurant levels attenuates lung function for at least one hour and augments cytokine production and carbohydrate utilization for at least three hours. In addition, we recently showed that SHS at levels equal to those adopted herein adversely affects the cardiorespiratory and immune response to prolonged submaximal physical exertion for at least three hours [28]. In the present study, we found that the cardiorespiratory and immune systems are also compromised when SHS is followed by brief physical exertion of maximal intensity. These results suggest that individuals who are exposed to SHS and then have to undergo maximal physical exertion will under-perform, perceive the required task as more difficult, and will be at an increased risk for cardiovascular, respiratory, or allergic symptoms.

Despite their decline in recent decades, the prevalence of smoking in blue-collar workers [i.e., individuals working in farming, fishing, forestry, construction, extraction, maintenance, production, transportation, and material moving occupations] [30] of developed countries remains higher than that of all other occupational groups combined [16], [17], [31], [32]. Moreover, blue collar workers have a higher risk for SHS at work [15], [33]. These statistics suggest that an enormous part of the population that are physically active on a daily basis for at least 8 hours are, at the same time, smoking passively. Indeed, blue collar workers account for 24.7% and 25.8% of the total workforce in the United States [30] and the European Union [34], respectively.

Based on the present results, SHS is associated with a 12% decrease in maximum power output, an 11.5% decrease in relative power output, an 8.2% reduction in maximal oxygen consumption, a 6% increase in perceived exertion, a 6.7% decrease in time to exhaustion, and a 9.8% increase in recovery systolic blood pressure at *T*_0_ (Tables 1, 2, 3). It is important to also note that perceived exertion and recovery systolic blood pressure remained elevated even when physical exertion was administered three hours following SHS. Lung function was markedly deteriorated when the physical task was administered immediately following SHS and tended to return to baseline values thereafter. Yet, _%_FEV_1_, FEV_1_/FVC, respiratory rate and tidal volume remained affected in women even three hours following SHS, suggesting that the effects of SHS on lung function are intensified in women when maximal physical exertion is involved. The observed SHS-induced increase in circulating inflammatory markers extends the findings of a small number of previous human experiments reporting SHS-induced increases of IL-1 beta, TNF-α [6], [28], white blood cell count, C-reactive protein, homocysteine, fibrinogen [11] as well as leukocyte counts accompanied by an activation of the immune cells [13]. The fact that IL-4, TNF-α, and IFN-γ remained elevated at *T*_3_ only in men supports recent experiments by our group [6], [28] showing an increased SHS-induced inflammatory reaction in men compared to women.

The only previous experiment investigating responses to maximal physical exertion following SHS reported prolonged time to heart rate recovery and decreased maximal oxygen uptake [35]. Unfortunately, this experiment incorporated very intense SHS (i.e., 30–35 ppm CO). A recent study reported that CO concentrations in Polish pubs can reach as high as 33 ppm, yet this very high concentration was observed only in 4.5% of the assessed establishments, that were characterized by very small capaciousness and limited ventilation [36]. Furthermore, the aforementioned study assessing responses to maximal physical exertion following SHS presented very limited information regarding the exercise stress and the measurements conducted. As such, it is very difficult to derive useful conclusions based solely on the findings of this single report. To the best of our knowledge, the present paper presents results from the first experiment to use a standardized experimental protocol [5], [6], [12], [28] with SHS set at levels similar to those of average bars/restaurants to simultaneously investigate the effects of SHS and their duration on the cardiorespiratory and immune response to maximal physical exertion in healthy nonsmokers.

The observed changes in cardiopulmonary function and inflammatory cytokines did not arise from extreme and/or prolonged SHS. The reported cotinine levels suggest moderate and brief SHS [37], confirming a successful simulation of a bar/restaurant smoking environment. Further, it is not possible that the comparatively long half-life of cotinine, the diurnal variation in lung function and inflammatory markers, or the investigators' expectations were reflected in our results. This is because the adopted design enabled data collection without SHS exposure (*T*_B_) as well as immediately following (*T*_0_), at one hour after (*T*_1_) and at three hours following SHS (*T*_3_), with the data being collected at the same time of the day and without the effect of previous or subsequent measurements since only one assessment was conducted per trial. Yet, our lung function results are limited by the impossibility of blinding our participants to SHS. However, suggestibility does not appear to underlie acute physiological responses to SHS [38]. Moreover, the present SHS-induced inflammatory reaction observed at *T*_0_ was similar to a previous study by our group that incorporated a repeated-measures randomized-block design with a sham SHS exposure [5], providing support to our protocol.

In conclusion, our randomized crossover experiment revealed that one hour of SHS at levels similar to those of bars/restaurants adversely affects the cardiorespiratory and immune response to maximal physical exertion in young healthy nonsmokers for at least three hours following SHS exposure.
