# Correction: Respiratory and Immune Response to Maximal Physical Exertion following Exposure to Secondhand Smoke in Healthy Adults

**DOI:** 10.1371/annotation/f2eb18a5-4dd2-49c1-95e9-2299402ee5a6

**Published:** 2012-04-09

**Authors:** Andreas D. Flouris, Giorgos S. Metsios, Andres E. Carrillo, Athanasios Z. Jamurtas, Polychronis D. Stivaktakis, Manolis N. Tzatzarakis, Aristidis M. Tsatsakis, Yiannis Koutedakis

There is an error in the third author's name. The correct spelling is Andres E. Carrillo. The correct citation is: Flouris AD, Metsios GS, Carrillo AE, Jamurtas AZ, Stivaktakis PD, et al. (2012) Respiratory and Immune Response to Maximal Physical Exertion following Exposure to Secondhand Smoke in Healthy Adults. PLoS ONE 7(2): e31880. doi:10.1371/journal.pone.0031880 

